# Systolic myocardial function measured by echocardiographic speckle-tracking and peak oxygen consumption in pediatric childhood cancer survivors—a PACCS study

**DOI:** 10.3389/fcvm.2023.1221787

**Published:** 2023-07-05

**Authors:** Britt Engan, Simone Diab, Henrik Brun, Truls Raastad, Ingrid Kristin Torsvik, Tom Roar Omdal, Fatemeh Zamanzad Ghavidel, Gottfried Greve, Ellen Ruud, Elisabeth Edvardsen, Elisabeth Leirgul

**Affiliations:** ^1^ Department of Clinical Science, University of Bergen, Bergen, Norway; ^2^ Department of Heart Disease, Haukeland University Hospital, Bergen, Norway; ^3^ Department of Pediatric Cardiology, Oslo University Hospital, Oslo, Norway; ^4^ The Intervention Centre, Technology and Innovation Clinic, Oslo University Hospital, Oslo, Norway; ^5^ Department of Sports Medicine, The Norwegian School of Sport Sciences, Oslo, Norway; ^6^ Department of Pediatric and Adolescent Medicine, Haukeland University Hospital, Bergen, Norway; ^7^ Centre for Clinical Research, Haukeland University Hospital, Bergen, Norway; ^8^ Department of Pediatric Medicine, Oslo University Hospital, Oslo, Norway

**Keywords:** pediatric childhood cancer survivors, myocardial function, speckle-tracking echocardiography, post-systolic shortening, peak oxygen consumption

## Abstract

**Background:**

Cancer therapy-related cardiotoxicity is a major cause of cardiovascular morbidity in childhood cancer survivors. The aims of this study were to investigate systolic myocardial function and its association to cardiorespiratory fitness in pediatric childhood cancer survivors.

**Methods:**

In this sub-study of the international study “Physical Activity and fitness in Childhood Cancer Survivors” (PACCS), echocardiographic measures of left ventricular global longitudinal strain (LV-GLS) and right ventricular longitudinal strain (RV-LS) were measured in 128 childhood cancer survivors aged 9–18 years and in 23 age- and sex-matched controls. Cardiorespiratory fitness was measured as peak oxygen consumption achieved on treadmill and correlated to myocardial function.

**Results:**

Mean LV-GLS was reduced in the childhood cancer survivors compared to the controls, −19.7% [95% confidence interval (CI) −20.1% to −19.3%] vs. −21.3% (95% CI: −22.2% to −20.3%) (*p* = 0.004), however, mainly within normal range. Only 13% of the childhood cancer survivors had reduced LV longitudinal strain *z*-score. Mean RV-LS was similar in the childhood cancer survivors and the controls, −23.2% (95% CI: −23.7% to −22.6%) vs. −23.3% (95% CI: −24.6% to −22.0%) (*p* = 0.8). In the childhood cancer survivors, lower myocardial function was associated with lower peak oxygen consumption [correlation coefficient (*r*) = −0.3 for LV-GLS]. Higher doses of anthracyclines (*r* = 0.5 for LV-GLS and 0.2 for RV-LS) and increasing time after treatment (*r* = 0.3 for LV-GLS and 0.2 for RV-LS) were associated with lower myocardial function.

**Conclusions:**

Left ventricular function, but not right ventricular function, was reduced in pediatric childhood cancer survivors compared to controls, and a lower left ventricular myocardial function was associated with lower peak oxygen consumption. Furthermore, higher anthracycline doses and increasing time after treatment were associated with lower myocardial function, implying that long-term follow-up is important in this population at risk.

## Introduction

Cardiovascular disease is a leading non-cancer contributor to early morbidity and mortality in childhood cancer survivors (CCSs) (1). In addition to the poorer cardiovascular risk profile observed in the CCSs compared to the general population (1), exercise intolerance and decreased maximal oxygen consumption, associated with increased all-cause mortality, are reported to be prevalent among adult CCSs (2). Exercise performance is also reported to be reduced in a few small studies of pediatric CCS populations (3–5).

Cancer treatment includes a variety of chemotherapeutic medication and radiation therapy, associated with dose-dependent cardiovascular toxicity (6). Younger age at the time of treatment and female sex have been associated with increasing treatment-related cardiotoxicity (1). However, not only cancer treatment but also the disease itself might be a risk factor for cardiovascular late effects due to chronic inflammation and oxidative stress present in all cancer diseases (7).

Despite the development of modern targeted therapies, anthracycline chemotherapy is used to treat a wide spectrum of childhood cancers (8). Anthracyclines belong to the class of chemotherapeutic drugs with most prominent association with cardiotoxicity (1) and are known to cause acute and late cardiomyopathy, often irreversible and progressive (6). Other classes of antineoplastic drugs, such as vinca alkaloids and platinum derivatives, have mainly been linked to cardiac ischemia, thromboembolism, and hypertension, but also heart failure (9).

Echocardiography continues to be the mainstay in the evaluation of myocardial function related to cancer treatment. Compared to traditional measures of myocardial function such as ejection fraction (EF) and fractional shortening, measures of speckle-tracking-derived strain have shown to be more sensitive to changes in left ventricular myocardial function and to provide additional prognostic information (10, 11). Former studies of myocardial function in CCSs measured by echocardiographic strain have shown to be more global than regional impairment of left ventricular function, and impairment of the right ventricle, suggesting a uniform effect of cancer and its treatments on the myocardium (11–13). Echocardiographic speckle-tracking allows measures of post-systolic shortening (PSS), which represents delayed myocardial contraction, in terms of longitudinal shortening, occurring after end-systole. PSS has been described in myocardial segments with contractile dysfunction due to ischemia and hypertrophy and is associated with increased risk of cardiovascular events in high-risk populations (14–16). PSS has also been observed in the myocardium of healthy and low-risk subjects, and even in this population, it is reported to be a predictor of cardiovascular morbidity and death (17, 18).

Peak oxygen consumption (VO_2_) is mainly limited by cardiac output (19), and reduced stroke volume is correlated to reduced myocardial function measured by strain (20). To date, myocardial function measured by strain and cardiorespiratory fitness have mainly been investigated in adult survivors of childhood cancer. However, the evaluation of cardiac function and fitness in pediatric CCSs is of importance to identify cardiovascular risk at an early point. The aim of the present study was to investigate biventricular myocardial function by echocardiographic measures of left (LV-LS) and right ventricular longitudinal strain (RV-LS) and PSS and to investigate the association between myocardial function and cardiorespiratory fitness measured by peak VO_2_, in pediatric CCSs. Our hypothesis was that myocardial function is reduced in pediatric CCSs compared to controls and associated with lower cardiorespiratory fitness.

## Materials and methods

### Study design and participants

This study was a sub-study of the international multicenter cohort study “Physical Activity and fitness in Childhood Cancer Survivors” (PACCS) (21) with main aims to identify the level of and mediators of activity and physical fitness in CCSs. Children and adolescents aged 9–18 years with childhood cancer treatment finished at least 1 year before were invited to participate in the study when attending routine follow-up at oncological outpatient clinics at their hospitals. Exclusion criteria were severe activity triggered arrhythmia, heart failure with echocardiographic measured fraction shortening (FS) below 20%, or physical function too poor to enable the completion of the physical tests. Healthy age- and sex-matched schoolmates were recruited as control participants.

In the present sub-study, CCSs from the pediatric cancer outpatient clinic at Oslo University Hospital (Oslo, Norway) and Haukeland University Hospital (Bergen, Norway) were invited to participate. Of the 207 eligible CCSs from the PACCS study, 66 declined the invitation and 13 withdrew after inclusion. No one was excluded due to heart failure, arrhythmia, or too low physical capacity. None of the participants used cardiac medication. A total of 128 CCSs underwent echocardiographic examination, and 126 underwent cardiopulmonary exercise test. Due to capacity challenges regarding echocardiographic imaging, only one center, Haukeland University Hospital, recruited controls, and 50 controls were invited. The participants were examined in the period from February 2019 to February 2021. Due to the ongoing COVID-19 pandemic with several restrictions regarding traveling and entrance to the hospitals, and frequent COVID-quarantine in school children, only 23 controls were included.

The CCSs had been treated for many different cancer forms and were categorized according to the International Classification of Childhood Cancer, third edition (ICCC3) (22). The cumulative doses of anthracyclines were calculated as equipotent doses of doxorubicin (mg/m^2^) according to the Children’s Oncology Group’s long-term follow-up guidelines version 5.0 (23) and Feijen et al. (24) (Table 1).

**Table 1:** Equipotent doses of doxorubicin.

Doxorubicin	Multiply total dose × 1
Daunorubicin	Multiply total dose × 0.5
Epirubicin	Multiply total dose × 0.67
Idarubicin	Multiply total dose × 5
Mitoxantrone	Multiply total dose × 4

Cumulative equipotent doses of doxorubicin (mg/m^2^) calculated according to the Children's Oncology Group Long-Term Follow-Up Guidelines version 5.0 (http://www.survivorshipguidelines.org/).

The study was conducted in accordance with the Declaration of Helsinki. The Regional Committee for Medical and Health Research Ethics of the South-East Norway Health Authority approved the study (REC 2018/739). Written and verbal information were given to the participants that were adapted to their age and developmental stage. Written informed consent was obtained from all participants and their parents or legal guardians.

### Echocardiographic assessment

All echocardiographic examinations were performed with the Vivid E9 ultrasound system (GE Healthcare, Horten, Norway) using a 4Vc-D (1.4–5.2 MHz) transducer for all imaging in Bergen and an equivalent 5Sc (1.5–4.6 MHz) transducer for all imaging in Oslo. Data were stored on external hard drives or Digital Versatile Discs allowing offline analyses with the echocardiographic software, EchoPAC version 204 (GE Healthcare, Horten, Norway). The participants were examined at rest in supine position. No sedation was used. All the participants underwent a comprehensive functional echocardiographic examination including grayscale images optimized for two-dimensional (2D) speckle-tracking analysis. Quantification of systolic left ventricular (LV) and right ventricular (RV) function was performed following the recommendations from the European and American Societies of Echocardiography (25, 26).

**Figure 1:**
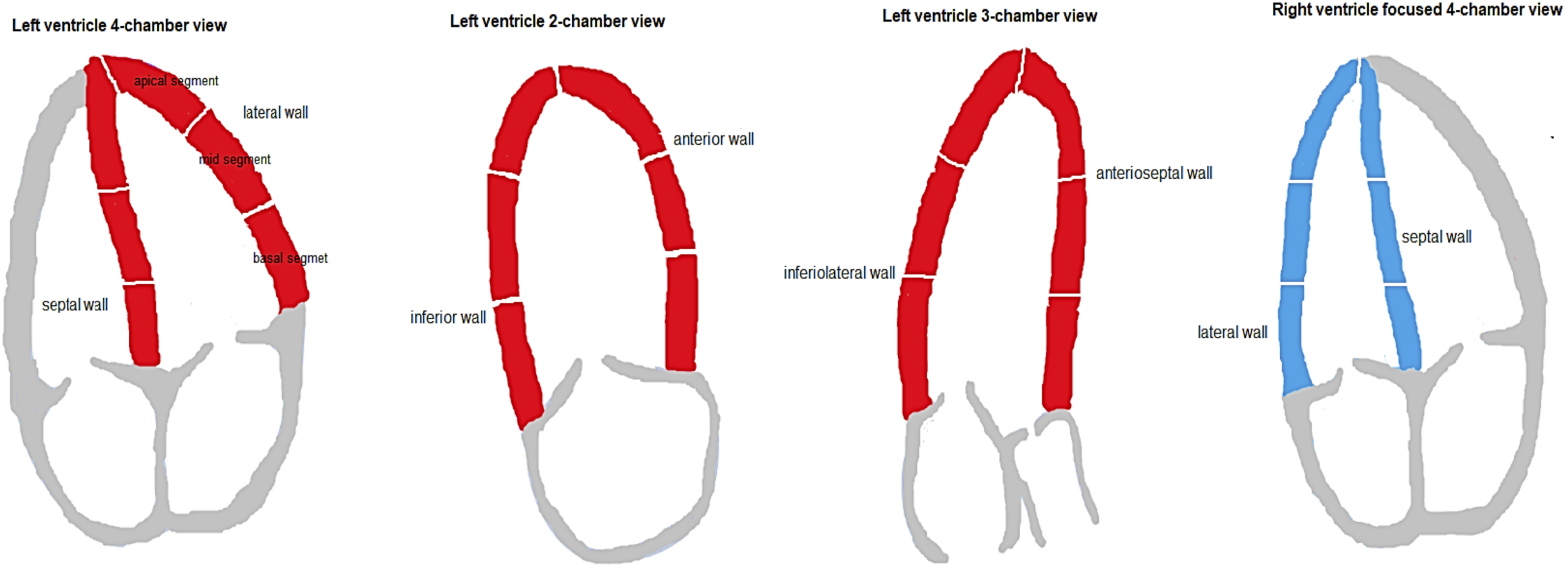
Left and right ventricular walls and wall segments.

LV-EF was calculated by the modified Simpson biplane technique. Grayscale images for 2D speckle-tracking analysis were acquired at frame rate (frames/second) to heart rate (beats/minute) ratio of 0.7–0.9. Longitudinal strain curves from standard apical four-, three-, and two-chamber view images, analyzed for 18 sub-segments, were used for LV longitudinal strain assessments. Longitudinal strain curves from an RV-focused four-chamber view image, analyzed for six sub-segments including the interventricular septum, were used for RV longitudinal strain assessments (Figure 1).

A single cardiac cycle considered to have the most optimal images was selected for analysis of LV and RV 2D strain (27, 28). The region of interest (ROI) was defined by manual tracing along the endocardial border from the atrioventricular valve annulus to the apex, and the thickness was adjusted to cover the myocardium without including the pericardium. Tracking was performed automatically. If necessary, the ROI was adjusted manually until the best possible tracking was obtained. Tracking compromised by artifacts/shadows, or image acquisitions with more than one faulty segment were excluded. Peak systolic strain was measured at aortic valve closure for LV measurements and at pulmonary valve closure for RV measurements. The timing of the aortic and pulmonary valve closure was assessed by continuous-wave Doppler.

In this paper, LV global longitudinal strain (LV-GLS) is used to describe the average peak systolic longitudinal strain from all three left ventricular apical views, and the term RV-LS is used to describe the peak systolic RV longitudinal strain from an RV-focused four-chamber view, including RV free wall and intraventricular septum. All longitudinal strain values are described as negative percentages, as it describes myocardial shortening of segments relative to the end-diastolic length.

LV-LS *z*-scores were calculated as proposed by Dallaire et al. (29) from measurements in apical four-chamber view images, normalized to body surface area (BSA) and adjusted for heteroscedasticity. The *z-*score calculation was based on imaging and analyses with similar echocardiographic ultrasound system and offline software as used in our study (29) and obtained from the equation [with BSA calculated by using the equation proposed by Haycock et al. (30)]

$$Z=\frac{\left(\mathrm{observed}\;\mathrm{value}-20.295\times \mathrm{BS}A^{-0.0614}\right)}{\left(-0.343\times \mathrm{BSA}+2.02\right)}$$

LV-LS *z*-score < −2 was considered abnormally low.

PSS, defined as late systolic longitudinal shortening appearing after the aortic valve closure in one cardiac cycle, was derived from calculations of post-systolic index (PSI). PSI was defined as longitudinal shortening after aortic closure relative to maximal global longitudinal strain:

$$\mathrm{PSI}=\frac{\left(\mathrm{peak}\;\mathrm{global}\;\mathrm{strain}-\mathrm{peak}\;\mathrm{systolic}\;\mathrm{strain}\right)}{\left(\mathrm{peak}\;\mathrm{global}\;\mathrm{strain}\right)}\times 100\text{\%}$$

**Figure 2:**
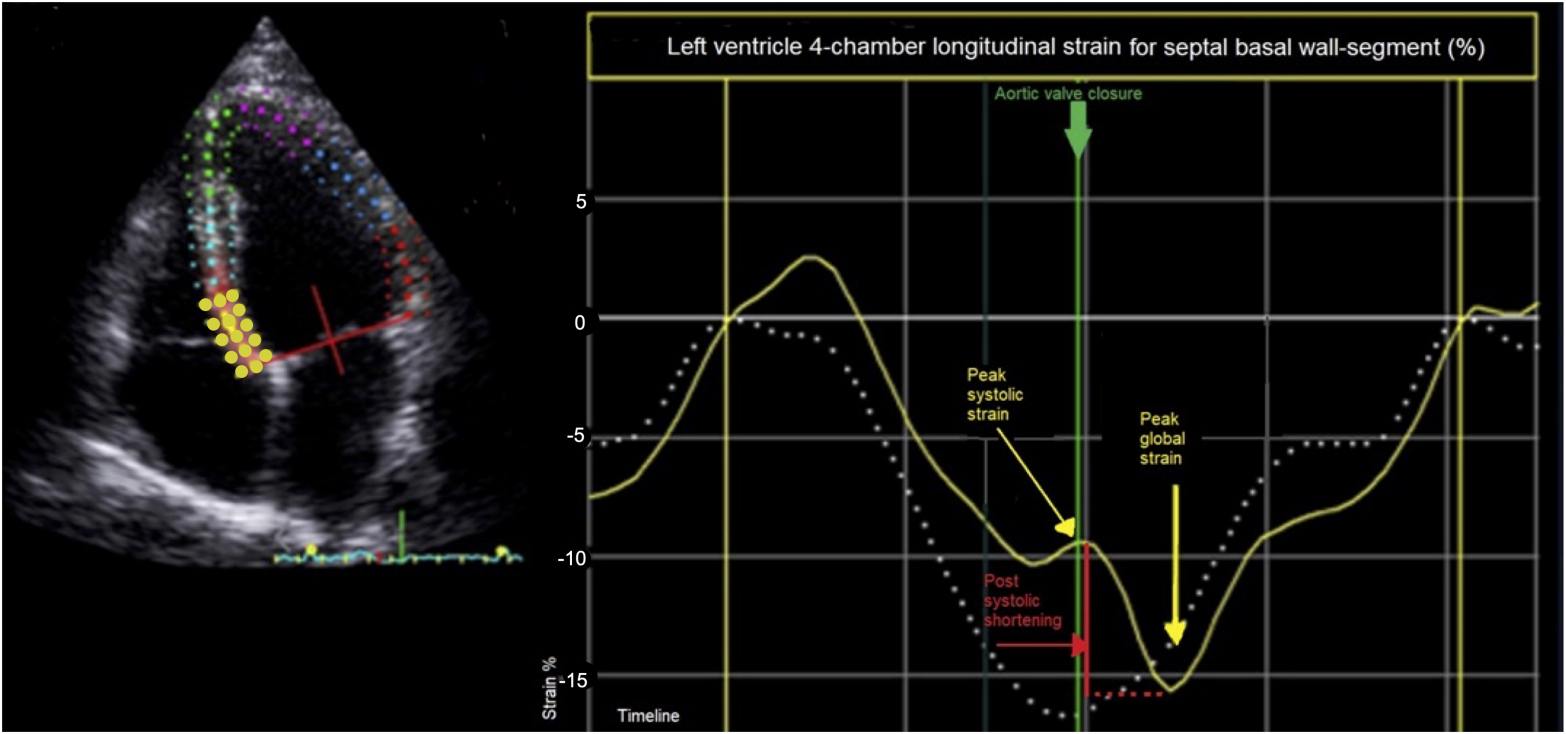
Speckle-tracking-derived longitudinal strain of basal septal wall segment (yellow line) in left ventricle four-chamber image. Post-systolic shortening (red line) is defined as late systolic longitudinal shortening appearing after the aortic valve closure (green line) in one cardiac cycle and is calculated as post-systolic index: [(peak global strain − peak systolic strain)/peak global strain] × 100%. In this example approximately {[−15% – (−10%)]/−15%} × 100% = 33%. The dashed white line represents longitudinal strain of a wall segment without post-systolic shortening.

If maximal global longitudinal strain was within the systole, PSI was set to zero. PSS was defined as PSI > 0% (Figure 2). For analysis, the average PSI of basal, mid, and apical LV wall segments, the average PSI of septal, lateral, anterior, inferior, anteroseptal, and inferolateral LV walls, and the average PSI of all LV and RV segments were used (Figure 1). The terms global LV PSI and global RV PSI were used to describe the average PSI of all LV and RV segments. Additionally, we registered the number of LV and RV wall segments with any degree of PSS.

All analyses were performed by a single experienced cardiologist (BE). Randomly selected study participants (5%) were obtained for inter-rater and intra-rater analyses. Another experienced cardiologist (TO) performed the re-analyses for inter-rater reliability. Both were blinded to the original results during re-analysis. Inter-rater reliability measured by a two-way mixed-effect model for absolute agreement was 0.8 [95% confidence interval (CI): 0.1–1.0] for EF Simpson, 0.9 (95% CI: 0.4–1.0) for LV-GLS, 0.9 (95% CI: 0.6–1.0) for RV-LS, and 0.9 (95% CI: 0.7–1.0) for global LV PSI. Intra-rater reliability measured by a two-way mixed-effect model for absolute agreement was 0.7 (95% CI: −0.3 to 0.9) for EF Simpson, 0.9 (95% CI: 0.5–1.0) for LV-GLS, 0.9 (95% CI: 0.6–1.0) for RV-LS, and 0.9 (95% CI 0.8–1.0) for global LV PSI.

### Cardiopulmonary exercise test

Cardiopulmonary exercise test was performed by walking/running on a treadmill (Woodway PPS MED, WOODWAY USA, Waukesha, WI, Unites States) with an incremental ramp protocol until exhaustion. The participant was breathing into a Hans Rudolph two-way breathing mask (2700 series; Hans Rudolph Inc., Kansas City, United States) connected to an OxyconPro analyzer (Jaeger, Würtzburg, Germany) using the breath-by-breath method. Borg scale (scale 6–20) (31) was used as rating of perceived exertion immediately after the test. The highest oxygen uptake measured, which remained stable over at least 30 s, was used as peak oxygen consumption (VO_2_) if two of the following three criteria were fulfilled: respiratory gas exchange ratio ≥1.10, Borg scale ≥17, or VO_2_ reached a plateau with increasing workload. The result for the CCSs was included in the present study to correlate peak VO_2_ with measures of myocardial systolic function.

### Anthropometry and blood pressure

Weight and standing height were measured with the participants wearing light clothing. Resting blood pressure was measured with an automated oscillometric device (in Bergen, Biolight BLT V6, Biolight Meditech Company, China; and in Oslo, Connex ProBP 3400, Welch Allyn, United States) after 5 min of rest in supine position, using the lowest of two measurements.

### Blood samples

Venous blood samples were collected from the CCSs only, in serum tubes and ethylenediaminetetraacetic acid-containing tubes, for measurements of hemoglobin (Hb) and N-terminal pro-brain natriuretic peptide (NT-proBNP). NT-proBNP values >160 ng/L were defined as above the upper limit in children aged 6–18 years (32).

### Statistical analyses

Descriptive variables and outcome data are presented as means with standard deviation (SD) or 95% CI, or as numbers and percentages. The Kolmogorov–Smirnov test was used to test the normality of the outcome variables, and Levene's test of equality of error variance was used to test the homogeneity of the outcome variables variance across groups. For comparison of descriptive variables and outcome data (without significant confounders) between CCSs and controls, Chi-square test or Fisher’s exact test, independent samples *t*-tests (with equal variance not assumed), and two-sample Kolmogorov–Smirnov test were applied as appropriate.

Linear regression analysis was used to identify confounders significantly affecting the analyses. Analyses of covariance (ANCOVA) were used to compare CCSs and controls for height, BSA, and BMI adjusted for sex; blood pressure adjusted for sex and age; EF and LS *z*-score adjusted for echocardiographic transducer difference; longitudinal strain adjusted for transducer difference and BSA; and peak VO_2_ adjusted for sex, age, and treadmill location.

All comparisons were re-analyzed without outliers, which did not significantly affect the results.

To examine whether the differences in EF, LV-GLS, and RV-LS between the CCSs and controls differed by sex, an interaction term for sex and group affiliation was added.

Comparisons of descriptive variables and outcome data between subgroups of study participants were done by chi-square test or Fisher`s exact test, Welch's ANOVA or classic one-way ANOVA (with Games–Howell and Tukey's test used for *post hoc* analyses, respectively), or by multiple comparison analyses (with the Sidak correction) with the same adjustments as described above, as appropriate.

The Pearson correlation coefficient (PCC) was used to explore associations between systolic myocardial function and treatment exposure, peak VO_2_ (adjusted for transducer/treadmill, sex, age, and hemoglobin concentration), age at diagnosis, and time after treatment, and between NT-proBNP and anthracycline dose or peak VO_2_.

All tests were two-sided, and *p* < 0.05 was considered statistically significant. All statistical analyses were performed using SPSS version 26.0 (IBM Corp., Armonk, NY, United States).

## Results

### Participant characteristics

Characteristics of the 151 study participants, 128 CCSs and 23 controls, are described in Table 2. The mean age was higher in the CCSs than in the control group, 13.6 years (SD 2.6) and 12.7 years (SD 3.1), respectively (*p *= 0.04), and mean systolic BP was lower in the CCSs compared to the controls, 107 vs. 112 mmHg, respectively (*p* = 0.03). The CCSs and controls were similar with regard to sex and ethnicity distribution, and estimated means of height, weight, BSA, BMI, and diastolic blood pressure.

**Table 2:** Characteristics of pediatric childhood cancer survivors and controls.

	CCS (*n* = 128)		Controls (*n* = 23)		*p*
	*n*		*n*		
Age at study, years, mean (SD)		13.6 (2.6)		12.7 (3.1)	0.04^a^
Sex, males	66	52%	10	44%	0.48^b^
Ethnicity, Caucasian	117	91%	23	100%	0.79^b^
Ethnicity, Asian	3	2%			
Ethnicity, mixed	7	6%			
Ethnicity, other	1	1%			
Height, cm, mean (95% CI)		158 (155–160)		153 (147–158)	0.11^c^
Weight, kg, mean (SD)		51 (15)		45 (14)	0.08^d^
BMI, kg/m^2^, mean (95% CI)		20.2 (19.6–20.9)		19.0 (17.5–20.5)	0.14^c^
BSA, m^2^, mean (95% CI)		1.5 (1.4–1.5)		1.4 (1.3–1.5)	0.09^c^
Systolic BP, mmHg, mean (95% CI)		107 (105–108)		112 (108–116)	0.03^c^
Diastolic BP, mmHg, mean (95% CI)		65 (64–67)		69 (65–73)	0.07^c^
Age at diagnosis, years, mean (SD)		5.3 (3.5)			
Time after treatment, years, mean (SD)		6.6 (3.6)			
ICCC3-1 (leukemias)	62	48%			
ICCC3-2 (lymphomas)	12	9%			
ICCC3-3 (CNS neoplasms)	17	13%			
ICCC3-4 (PNS tumors)	8	6%			
ICCC3-5 (retinoblastoma)	2	2%			
ICCC3-6 (renal tumors)	14	11%			
ICCC3-7 (hepatic tumors)	1	1%			
ICCC3-8 (malignant bone tumors)	6	5%			
ICCC3-9 (extraosseous sarcomas)	6	5%			
Chest radiation, Gy, mean (SD)	6	20 (9)			
Chemotherapy	124	97%			
Anthracyclines	97	78%			
Anthracycline dose, mg/m^2^, mean (SD)	95	152 (91)			
Anthracycline dose <100 mg/m^2^, mg/m^2^, mean (SD)	36	79 (5.8)			
Anthracycline dose 100–250 mg/m^2^, mg/m^2^, mean (SD)	44	152 (38)			
Anthracycline dose 250–500 mg/m^2^, mg/m^2^, mean (SD)	15	329 (63)			
Vinca alkaloids	115	93%			
Vincristine dose, mg/m^2^, mean (SD)	110	28 (13)			
Vinblastine dose, mg/m^2^, mean (SD)	3	144 (148)			
Vinorelbine dose, mg/m^2^, mean (SD)	1	706			
Platinum derivatives	28	23%			
Cisplatin dose, mg/m^2^, mean (SD)	13	289 (142)			
Carboplatin dose, mg/m^2^, mean (SD)	24	3,639 (294)			
NT-proBNP, ng/L, mean (SD)		67 (48)			
Hb, g/dl, mean (SD)		13.5 (1.0)			

BMI, body mass index; BP, blood pressure; CNS, central nervous system; PNS, peripheral nervous system.

^a^ Non-parametric two-sample Kolmogorov–Smirnov test.

^b^ Chi-square test or Fisher’s exact test.

^c^ ANCOVA adjusted for sex and age as appropriate.

^d^ Independent samples *t*-test (with equal variance not assumed).

For the CCSs, the mean age at diagnosis was 5.3 years (SD 0.3), and mean time after treatment was 6.6 years (SD 3.6). The most frequent diagnosis in the cancer survivor group was acute leukemia (48%), cerebrospinal neoplasms (13%), and renal tumors (11%). Most participants received multiple chemotherapeutic agents during the treatment period, detailed in Table 2. The majority, 78%, was treated with anthracyclines, with a mean dose of 152 mg/m^2^ (SD 91), while 22% were anthracycline naive and treated with vinca alkaloids and/or platinum derivatives. Six (5%) CCSs underwent radiation therapy involving the chest, all of them had also received chemotherapy. Four (3%) had surgical treatment, but no chemotherapy or radiation therapy (all diagnosed with cerebrospinal neoplasms).

Four CCSs had NT-proBNP values >160 ng/L defined as the upper limit in children aged 6–18 years (32), and mean NT-proBNP was 67 ng/L (SD 48).

### Left ventricle systolic function measured by EF

The CCSs had significantly lower mean EF Simpson compared to the controls (Table 3). Mean EF Simpson was 60% (95% CI: 59%–61%) for CCSs and 64% (95% CI: 61%–66%) for controls (*p* = 0.01).

**Table 3:** Measures of myocardial systolic function and peak oxygen consumption in childhood cancer survivors and controls.

	CCS		Controls		*p*
	*n*	Mean (95% CI)	*n*	Mean (95% CI)	
EF Simpson, %	128	60 (59 to 61)	23	63 (61 to 66)	0.01^a^
LV-4C-LS, %	126	−19.3 (−19.7 to −19.0)	23	−21.2 (−22.1 to −20.2)	<0.001^b^
*z*-score LV-LS	126	−0.3 (−0.6 to −0.1)	23	0.9 (0.2 to 1.5)	0.001^a^
*z*-score < −2	16	−2.7 (−3.1 to −2.4)	0		
LV-3C-LS, %	118	−19.6 (−20.0 to −19.1)	22	−20.5 (−21.7 to −19.4)	0.13^b^
LV-2C-LS, %	124	−20.1 (−20.6 to −19.7)	22	−22.2 (−23.3 to −21.1)	0.001^b^
LV-GLS, %	118	−19.7 (−20.1 to −19.3)	22	−21.3 (−22.2 to −20.3)	0.004^b^
PSI-global LV, %	104	2.1 (1.5 to 2.4)	21	1.8 (1.0 to 2.5)	0.35^c^
PSI-LV-lateral wall, %	122	3.2 (2.62 to 4.06)	22	3.3 (1.5 to 5.2)	0.89^a^
PSI-LV-septal wall, %	126	2.0 (1.5 to 2.4)	22	1.9 (0.9 to 3.0)	0.98^a^
PSI-LV-anterior wall, %	117	1.2 (0.8 to 1.6)	21	0.5 (−0.5 to 1.4)	0.18^a^
PSI-LV-inferior wall, %	124	1.2 (0.9 to 1.6)	21	1.2 (0.3 to 2.0)	0.84^a^
PSI-LV-inferolateral wall, %	114	3.5 (2.6 to 4.3)	21	3.1 (0.9 to 5.2)	0.73^a^
PSI-LV-anteroseptal wall, %	117	1.6 (1.1 to 2.1)	21	1.7 (0.4 to 2.9)	0.94^a^
PSI-LV-all basal segments, %	114	2.9 (2.4 to 3.4)	21	1.7 (0.4 to 3.0)	0.11^a^
PSI-LV-all mid segments, %	118	1.2 (0.9 to 1.4)	21	0.7 (0.1 to 1.3)	0.20^a^
PSI-LV-all apical segments, %	108	2.4 (1.8 to 2.9)	21	3.0 (1.6 to 4.3)	0.46^a^
LV-segments with PSI > 0%, %	126	41%	22	33%	0.003^c^
RV-LS, %	121	−23.2 (−23.7 to −22.6)	23	−23.3 (−24.6 to −22.0)	0.84^b^
PSI-global RV, %	103	1.8 (1.2 to 2.4)	18	2.3 (0.7 to 3.8)	0.59^a^
RV segments with PSI > 0%, %	120	30%	21	21%	0.04^c^
Peak VO_2_, ml/kg/min	126	43.2 (41.4 to 44.9)	22	48.6 (44.5 to 52.6)	0.01^d^

LV-4C-LS, left ventricular four-chamber longitudinal strain; LV-3C-LS, left ventricular three-chamber longitudinal strain; LV-2C-LS, left ventricular two-chamber longitudinal strain.

^a^ ANCOVA adjusted for transducer-dependent differences.

^b^ ANCOVA adjusted for BSA and transducer-dependent differences.

^c^ Chi-square test.

^d^ ANCOVA adjusted for age, sex, and treadmill location-dependent differences.

The difference in EF Simpson between CCSs and controls was not significantly greater in female vs. male participants: 2.7% (95% CI: −1.7% to 7.1%) (*p* = 0.23).

Left and right ventricular systolic function measured by longitudinal strain due to poor acoustic conditions measurements of LV-GLS and RV-LS were excluded in 11 and 7 participants, respectively.

**Figure 3:**
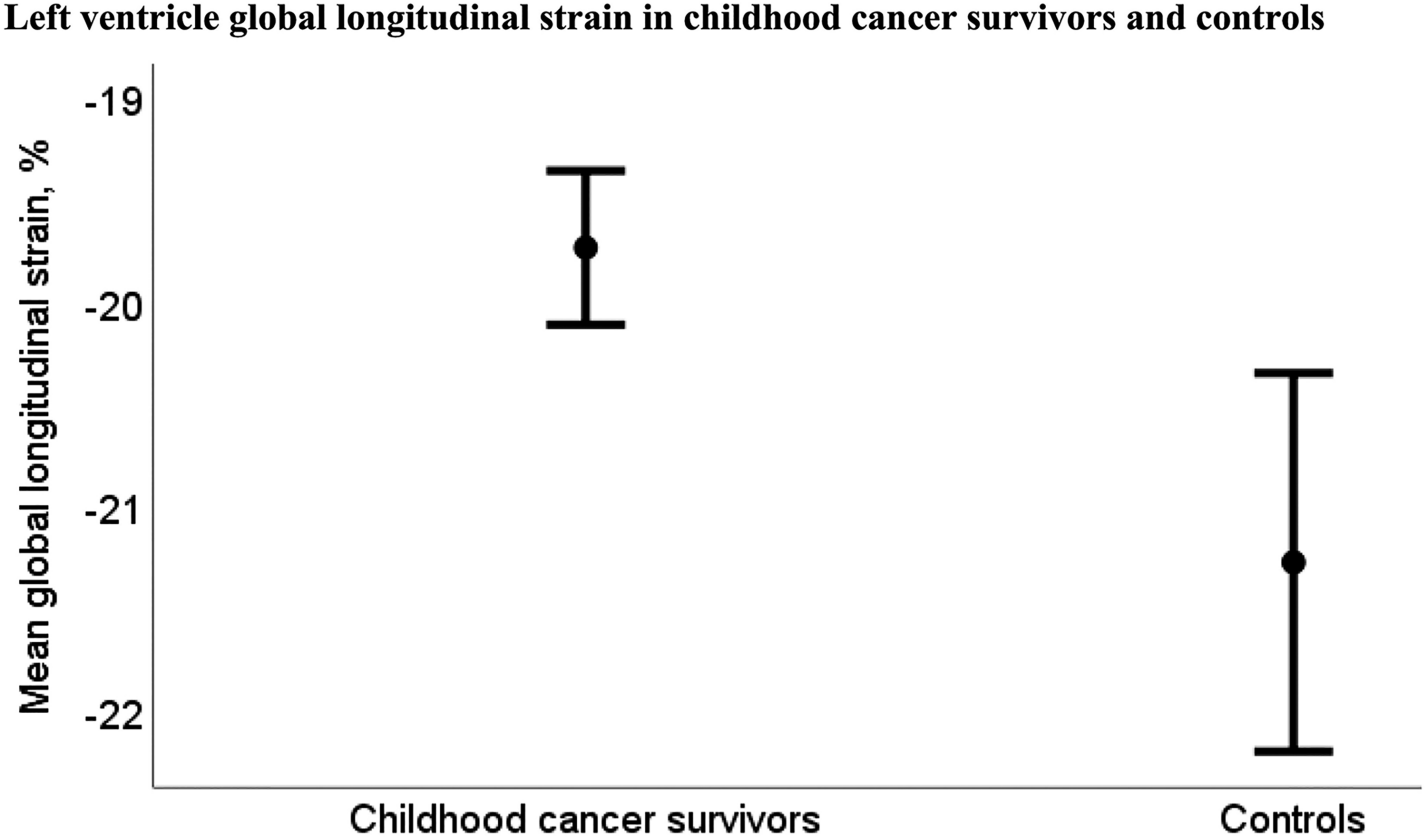
Mean left ventricular global longitudinal strain was −19.7% (95% CI −20.1% to −19.3%) for childhood cancer survivors (*n* = 118) and −21.3% (95% CI: −22.2% to −20.3%) for controls (*n* = 22) (*p* = 0.004).

The CCSs had significantly reduced mean LV-GLS compared to the controls, while mean RV-LS did not differ significantly between CCSs and controls (Table 3). Mean LV-GLS was −19.7% (95% CI: −20.1% to −19.3%) for CCSs and −21.3% (95% CI: −22.2% to −20.3%) for controls (*p* = 0.004) (Figure 3) and mean RV-LS was −23.2% (95% CI: −23.7% to −22.6%) for CCSs and −23.3% (95% CI: −24.6% to −22.0%) for controls (*p* = 0.8)

The difference in LV-GLS and RV-LS between CCSs and controls was not significantly greater in female vs. male participants, −0.3% (95% CI: −2.1% to 1.5%) (*p* = 0.74) and 2.1% (95% CI: −0.6% to 4.8%) (*p* = 0.12), respectively.

**Figure 4:**
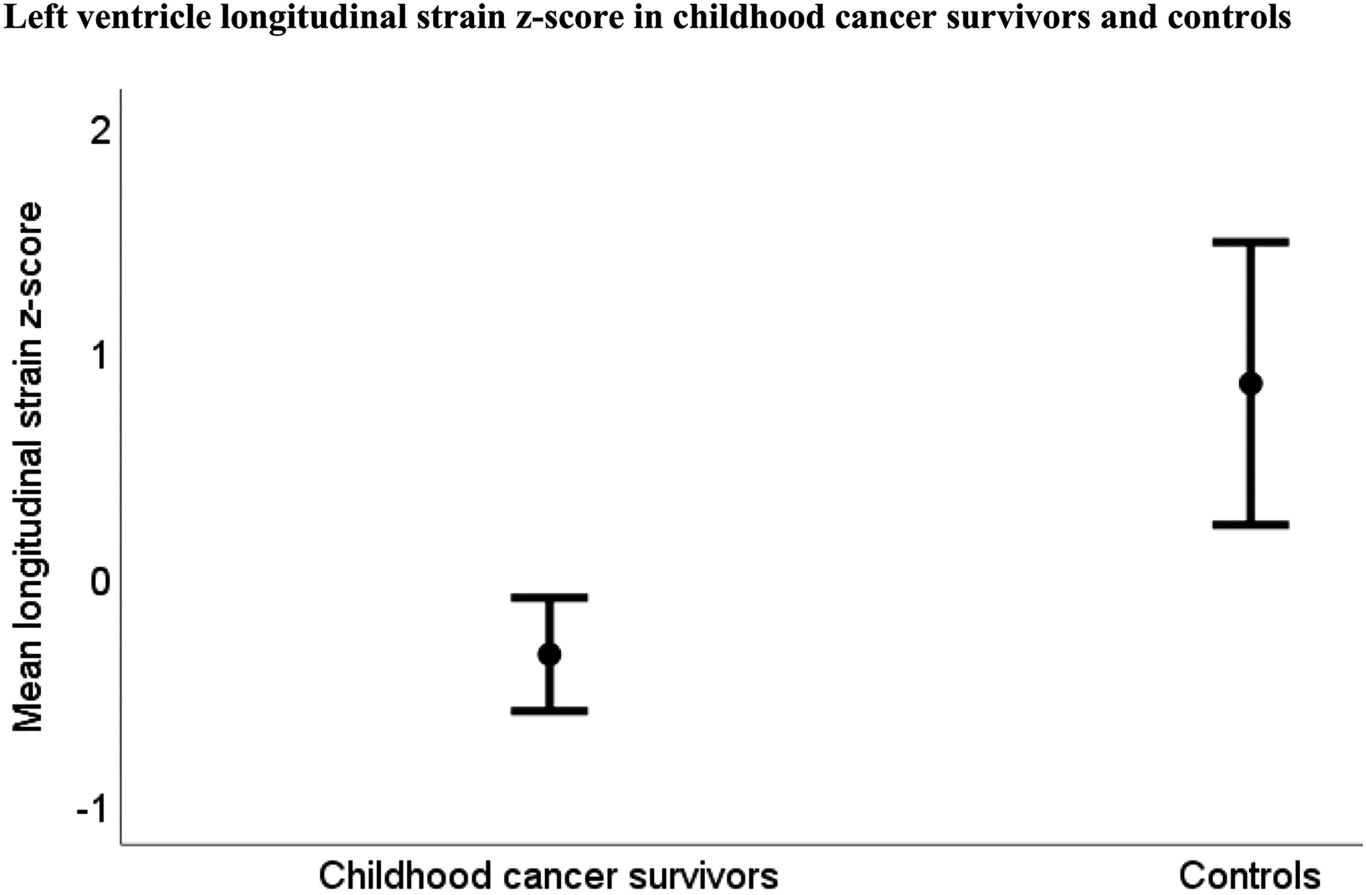
Mean left ventricular longitudinal *z*-score was −0.3 (95% CI −0.6 to −0.1) in childhood cancer survivors (*n* = 118) and 0.9 (95% CI: 0.2–1.5) in controls (*n* = 22) (*p* = 0.001).

None of the controls, but 16 (13%) of the CCSs, had abnormally low LV-LS *z*-score < −2, and mean LV-LS *z*-score was reduced in the CCSs compared to the controls (Table 3 and Figure 4). The mean LV-LS *z*-score was −0.3 (95% CI: −0.6 to −0.1) for CCSs and 0.9 (95% CI: 0.2 to 1.5) for controls (*p* = 0.001).

Increasing time after treatment was associated with lower LV and RV systolic function with PCC 0.3 for LV-GLS (*p* = 0.004) and 0.2 for RV-LS (*p* = 0.02). Adjustment for anthracycline dose did not alter the result. We found no significant association between age at diagnosis, NT-proBNP, and the measurements of LV and RV systolic function.

Post-systolic shortening, the presence of any degree of PSS (defined as PSI > 0%) was found more frequently in the wall segments of CCSs compared to the wall segments of controls (Table 3).

Any degree of PSS was found in 41% (904 of 2,192) of all the LV wall segments in CCSs and in 33% (127 of 384) of all the LV wall segments in the controls (*p* = 0.003), and in 30% (213 of 700) of all the RV wall segments in the CCSs and 21% (26 of 123) of all RV wall segments in the controls (*p* = 0.04).

Mean global LV and RV PSI, and PSI in LV walls and regional wall segments were similar in CCSs and controls (Table 3). Mean global LV PSI was 2.1% (95% CI: 1.5%–2.4%) for CCSs and 1.8% (95% CI: 1.0%–2.5%) for controls (*p* = 0.4), and mean global RV PSI was 1.8% (95% CI: 1.2%–2.4%) for CCSs and 2.3% (95% CI: 0.7%–3.8%) for controls (*p* = 0.6).

### Chemotherapeutic agents and systolic myocardial function and NT-proBNP

Exposure to increasing doses of anthracyclines was associated with lower systolic myocardial function. PCC for EF Simpson was −0.2 (*p* = 0.03), for LV-GLS was 0.5 (*p* < 0.01), and for RV-LS was 0.2 (*p* = 0.04). Correspondingly, NT-proBNP levels were positively associated with anthracycline dose with PCC 0.4 (*p* < 0.001). There was no significant association between increasing doses of vincristine, cisplatin, or carboplatin and measures of systolic LV or RV function.

### Radiation therapy and systolic myocardial function

Radiation therapy to the chest was associated with lower LV systolic function measured by EF Simpson and LV-GLS. However, in analysis adjusted for anthracycline doses, there was no significant association between radiation therapy and LV systolic function.

### Peak oxygen consumption in the CCSs

**Figure 5:**
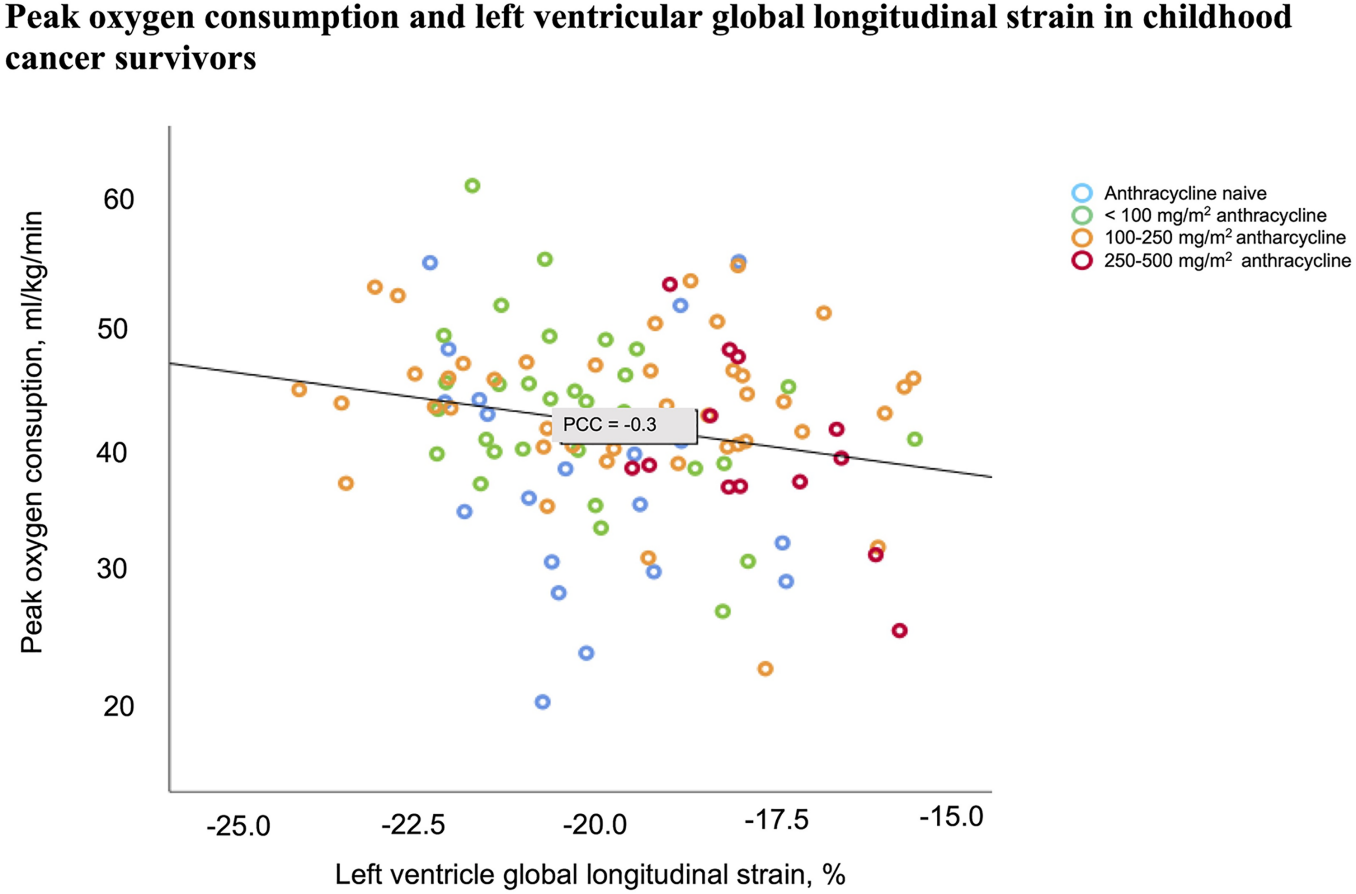
Decreasing myocardial function measured by left ventricular global longitudinal strain was associated with decreasing peak oxygen consumption in childhood cancer survivors (PCC −0.3, *p* = 0.01). The analysis was adjusted for transducer/treadmill location, sex, age, and hemoglobin concentration.

Mean peak VO_2_ was 43.2 ml/kg/min (95% CI 41.4–44.9) in CCSs and 48.6 ml/kg/min (95% CI: 44.5–52.6) in the controls (*p* = 0.01). Correlation analysis adjusted for sex, age, hemoglobin concentration, and transducer difference/treadmill location revealed a significant association between peak VO_2_ and LV function measured by LV-GLS (PCC −0.3, *p* = 0.01) (Figure 5) and EF Simpson (PCC 0.3, *p* = 0.002) in the CCSs, but no significant association between peak VO_2_ and RV-LS or NT-proBNP level in the CCSs.

### Subgroup analyses

We compared four subgroups of CCSs that were either anthracycline naive, had received low dose (<100 mg/m^2^), medium dose (100–250 mg/m^2^), or high dose (250–500 mg/m^2^) of anthracyclines (Supplementary Table S1). All four CCS subgroups included participants who had been treated with vinca alkaloids and/or platinum derivatives. The subgroups were similar regarding age at examination time, sex and ethnicity distribution, BSA, BMI, resting blood pressure, time since diagnosis, and time after treatment.

Mean LV-GLS, and corresponding LV-LS *z*-scores, were significantly reduced in the subgroup treated with high dose of anthracyclines [−18.0% (95% CI: −19.0% to −17.0%)] compared to the anthracycline naive subgroup [−20.3% (95% CI: −21.1% to −19.4%)], *p* = 0.003, and the subgroups treated with low dose [−20.3% (95% CI: −20.9% to −19.6%)], *p* = 0.001, and medium dose of anthracyclines [−19.7% (95% CI: −20.4% to −19.1%)], *p* = 0.01, as described in Supplementary Table S1. Correspondingly, the mean NT-proBNP level was significantly higher in the subgroup treated with high dose of anthracyclines [97 ng/L (SD 55)] compared to the anthracycline naive subgroup [54 ng/L (SD 30)], *p* = 0.05, and the subgroup treated with low dose of anthracyclines [52 ng/L (SD 24)], *p* < 0.03. NT-proBNP was also higher in the subgroup treated with medium dose of anthracyclines [71 ng/L (SD 36)] compared to the subgroup treated with low dose of anthracyclines (*p* = 0.03). RV-LS, global LV PSI, and global RV PSI were similar in all four CCSs subgroups.

**Figure 6:**
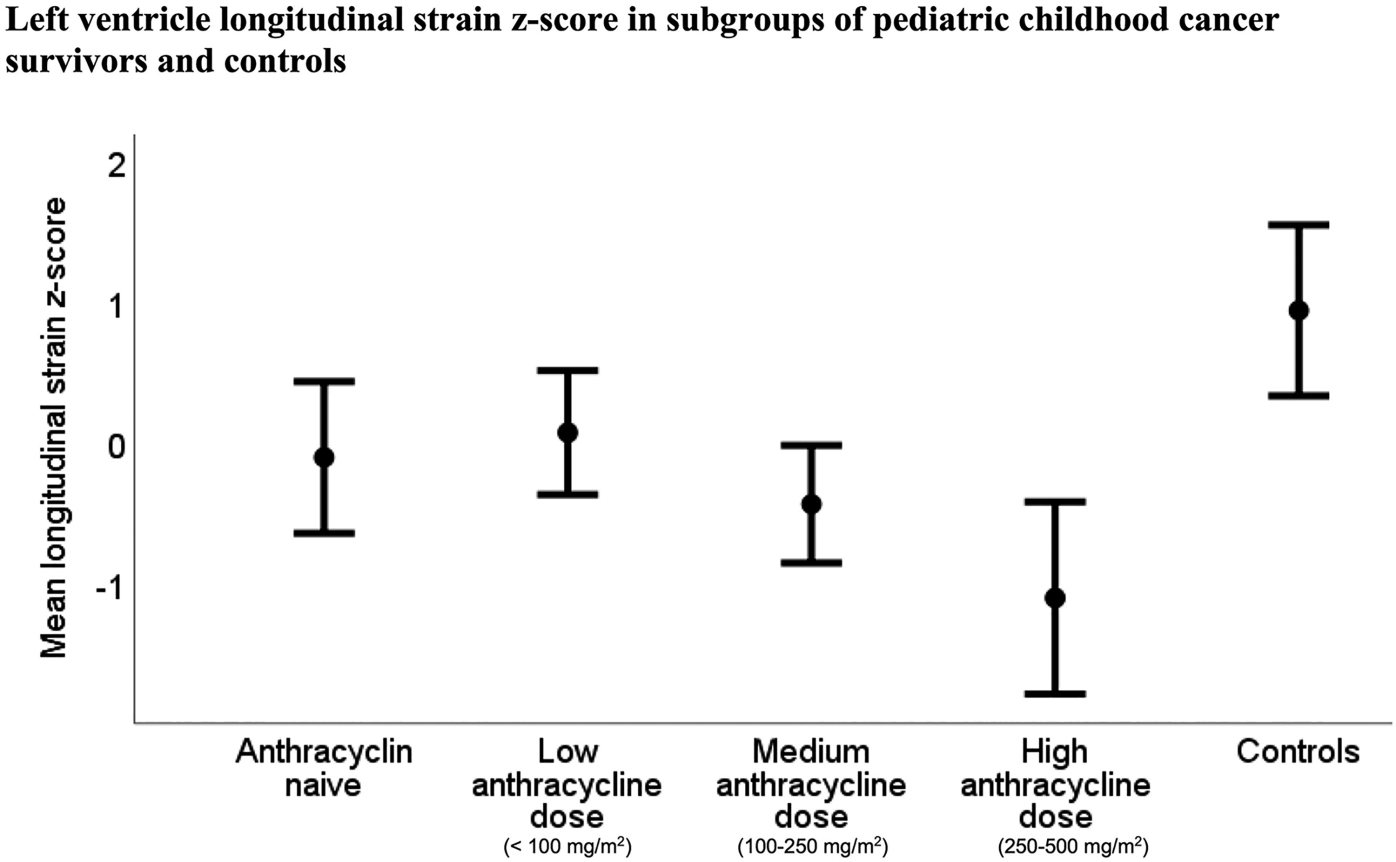
Mean LV-LS *z*-score was reduced in all of the four CCS subgroups compared to the control group (all *p*-values ≤ 0.02). For the anthracycline naive subgroup and the subgroups treated with low, medium, and high doses of anthracyclines, *z*-score was −0.1 [95% CI −0.6 to 0.4], 0.1 (95% CI: −0.4 to 0.5), −0.4 (95% CI: −0.8 to −0.02), and −1.1 (95% CI: −1.8 to −0.4), respectively, vs. 0.9 (95% CI: 0.2–1.5) in the controls.

**Figure 7:**
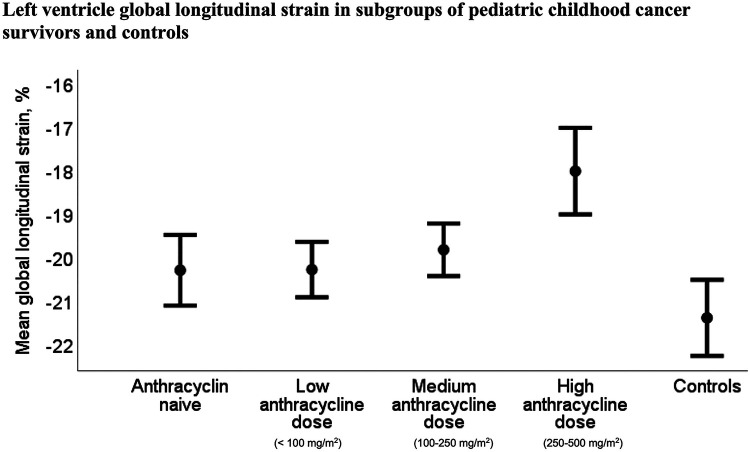
LV-GLS was significantly reduced in the childhood cancer survivor subgroups treated with high and medium dose of anthracyclines compared to the control group (*p*-values < 0.001 and *p* = 0.03, respectively). LV-GLS was significantly reduced in the childhood cancer survivor subgroup treated with high dose of anthracyclines [−18.0% (95% CI −19.0 to −17.2)] compared to the anthracycline naive subgroup [−20.3% (95% CI: −21.1 to −19.4)] and the subgroups treated with low dose [−20.3% (95% CI: −20.9 to −19.6)] and medium dose of anthracyclines [−19.7% (95% CI: −20.4 to −19.1)], *p* = 0.003, *p* = 0.01, and *p* = 0.001, respectively.

*z*-score for LV-LS was lower in all four CCSs subgroups compared to the control group (all *p*-values ≤ 0.02) (Figure 6), and mean LV-GLS was reduced in the two CCSs subgroups, which received medium and high dose of anthracyclines compared to the control group (both *p*-values ≤ 0.03) (Figure 7). RV-LS, global LV PSI, and global RV PSI were similar in all four CCSs subgroups compared to the control group.

## Discussion

In this multicenter study, we found lower LV, but not RV, systolic function in 128 pediatric CCSs, compared to 23 healthy controls. The systolic LV function in the CCSs was, however, largely within normal range, and only 13% of the CCSs had abnormally low LV strain *z*-score. Treatment with higher doses of anthracyclines and increasing time after treatment were associated with lower LV and RV systolic function, and lower LV myocardial function was associated with lower peak oxygen consumption during the cardiopulmonary exercise test.

Our finding of reduced LV-GLS in pediatric CCSs compared to controls indicates a detectable increased risk of developing heart failure in CCSs even at young age, and corresponds well to former findings in studies by Akam-Venkata et al. (11), Slieker et al. (13), and Yu et al. (33). The LV-LS *z*-score was <−2 in 13% of the CCSs, similar to the study by Slieker et al., which reported impaired LV longitudinal strain in 8% of 510 pediatric CCSs treated with similar mean anthracycline dose (150 mg/m^2^) as our study participants (13).

Our subgroup and correlation analyses revealed an inverse association between myocardial function measured by strain and anthracycline dose. This is in agreement with former knowledge of dose-dependent cardiovascular toxicity of cancer treatment (6). Worth noting is, however, that LV function measured by LV-LS *z*-score was found reduced also in the anthracycline naive CCS participants, and in the subgroup treated with low doses of anthracyclines, compared to controls. This supports former reports of associations between vinca alkaloids or platinum derivatives with heart failure (9, 34), and that there is probably no safe dose of anthracyclines (35). This might indicate that long-term follow-up including echocardiographic evaluation of myocardial function should be considered in all CCSs, and not only in the subjects treated with moderate to high doses of potentially cardiotoxic chemotherapeutic agents (36).

While previous studies have reported global myocardial impairment after cancer disease and cardiotoxic treatment (11, 12, 37, 38), we found similar RV-LS in CCSs and controls. However, there was an association between increasing time after treatment and more reduced both LV-GLS and RV-LS, and we could suspect that the cardiotoxic effect might progress and cause RV dysfunction later in life. This is consistent with previous reports of progressive myocardial toxicity and reports of reduced RV function in adult CCSs, but not in pediatric CCSs (12, 13, 39).

Younger age at time of cancer treatment and female sex have been associated with reduced myocardial function in CCSs, but we found no such associations. This may be due to a relative moderate mean dose of anthracyclines (152 mg/m^2^) in our study. Kremer et al. (40) summarized 10 studies from the late 90s to the early 2000s where the CCSs had been treated with mean or median anthracycline doses mainly above 250 mg/m^2^. Of these, two studies reported an association between younger age at the time of treatment and decreased myocardial function while four studies found the same association with female sex.

To further evaluate the cardiovascular risk and myocardial function in CCSs, we investigated PSS in the myocardial wall segments. Even though mean global PSI for both LV and RV were similar in CCSs and controls, we found a higher share of wall segments with any degree of PSS in the CCSs compared to the controls. The mechanisms behind the finding of increased presence of PPS in the CCSs are uncertain but might be due to myocardial micro infarction related to both the cancer disease and/or its treatment. Even though any degree of PSS has been found in approximately 30% of LV wall segments in healthy young adults (14), PSS has previously been reported to be a predictor of cardiovascular morbidity and death (17, 18). Our finding indicates that PSS might add as a marker of increased cardiovascular risk in the CCSs. However, further studies are needed to establish the clinical utility and additive value of measures of PSS in CCSs.

Similar to Wolf et al. (41), we found a positive association between anthracycline dose and NT-proBNP levels. However, even though the anthracycline dose was positively associated with NT-proBNP level and inversely associated with myocardial function, we found no significant association between NT-proBNP level and myocardial function. This is in agreement with former studies of NT-proBNP levels and myocardial function in CCSs (13, 42). However, as in our study, these studies included participants with mainly normal values of NT-proBNP. With the present cut-off values, diagnostic value of this biomarker to detect myocardial dysfunction in CCSs have been reported to be limited (43).

Only a few previous studies have investigated the relationship between myocardial function and the cardiorespiratory fitness in pediatric CCSs. Hogarty et al. (5) reported lower peak VO_2_ in 33 pediatric CCSs, while De Caro et al. (44) found similar peak VO_2_ in 84 pediatric CCSs compared to controls. The participants in both studies had mainly normal myocardial function measured by echocardiographic fractional shortening. Other studies have reported reduced peak VO_2_ in pediatric CCSs with reduced cardiac function revealed by myocardial imaging during exercise (4, 45), but also in CCSs with preserved myocardial stress response (4). Our study population is part of a larger study population included in the PACCS study where 157 pediatric CCSs have participated in cardiopulmonary exercise testing (46). Bratteteig et al. reported a positive association between physical activity levels and peak VO_2_ in these participants (46). In our sub-study, we found that lower LV myocardial function measured by EF Simpson and LV-GLS was associated with lower peak VO_2_. Although complex mechanisms may have contributed to this association, it could be suggestive of reduced cardiac reserve in the CCSs. The finding corresponds well with former findings in adult CCSs where exercise intolerance and decreased myocardial function are described as prevalent (2). These characteristics are associated with increased mortality (2) and are, therefore, important to identify in young CCSs. Additionally, as suggested by others, exercise testing might be useful to identify CCSs with subclinical myocardial impairment (41).

The strengths of this study are the cohort study design and that all echocardiographic imaging was performed by highly trained sonographers with the same ultrasound system, and all offline analyses were made by one cardiologist with the same echocardiographic software. The calculated intra- and inter-rater variabilities were good to excellent (47). The limited number of controls is the main limitation of the study and might increase the risk of type 1 error. For the main outcome variables (LV-GLS, RV-LS, and peak VO_2_), the homogeneity of variance was not violated. Therefore, we consider the risk of type 1 error to be small. There is a risk of selection bias since extensive and physically challenging testing was part of the main study. This may have led to a selection of the fittest CCSs, which could most likely introduce an overestimate of LV or RV function in the CCSs group. EF and strain measurements are known to be afterload dependent with more reduced EF and longitudinal strain with higher blood pressure (48). We found significantly higher systolic blood pressure in the controls compared to the CCSs, which may have led to an underestimate of the difference in systolic myocardial function between CCSs and controls in this study. Peak VO_2_ is mainly limited by the ability of the cardiovascular system to deliver oxygen to the exercising muscles (19), but other factors, such as respiratory or musculoskeletal dysfunction, deconditioning, or lack of motivation may also influence on the result. Cancer treatment is associated with multiple adverse effects that might have influenced on the peak VO_2_ results. Thus, complex mechanisms could have contributed to the association between decreasing myocardial function and decreasing peak oxygen consumption in the CCSs. Regression analyses revealed significant influence of location in the echocardiographic measures and peak VO_2_ results in the CCSs examined in Oslo vs. those examined in Bergen, which could not be explained by difference in chemotherapy doses, radiation therapy, blood pressure, or other characteristics. There might be technical- or procedure-related differences in exercise test results, as well as differences related to the ultrasound transducers used for echocardiographic imaging at the two centers. To address this issue as well as the single center inclusion of controls, correction for location was added in the statistical analyses.

Although the myocardial function was largely within normal range, we found reduced left ventricular systolic myocardial function in pediatric CCSs compared to healthy controls even in participants treated with low doses of anthracyclines. Lower left ventricular function was associated with lower peak VO_2_. The right ventricular function was similar in CCSs and controls suggesting regional differences regarding the impact of the cardiotoxic treatment on myocardial function. Increasing dose of anthracyclines and increasing time after treatment were, however, associated with lower myocardial function of both the left and right ventricles. This implies that long-term follow-up, including evaluation of myocardial function, and tight control of modifiable cardiovascular risk factors might be indicated for all childhood cancer survivors.

## Data Availability

The raw data supporting the conclusions of this article will be made available by the authors, without undue reservation.
