# Supplementary material for: Systolic myocardial function measured by echocardiographic speckle-tracking and peak oxygen consumption in pediatric childhood cancer survivors—a PACCS study
Source: Front Cardiovasc Med. 2023 Jul 5;10:1221787. doi: 10.3389/fcvm.2023.1221787 (PMC10354364; doi:10.3389/fcvm.2023.1221787)
Supplement: Supplementary file 1 [file Datasheet1.pdf]

Table 4: Characteristics and measures of myocardial systolic function and peak oxygen consumption in childhood cancer survivors by subgroups

	**CCSs Anthracycline naive** n=26	**CCSs Anthracycline dose <100mg/m^2** n=36	**CCSs Anthracycline dose 100-250mg/m^2** n=44	**CCSs Anthracycline dose 250-500mg/m^2** n=15	**p-value**	**Controls** n=23
Sex, males, n (%)	16 (62%)	16 (44%)	24 (55%)	6 (40%)	0.53[a]	10 (43%)
Ethnicity, Caucasian, n (%)<br>Ethnicity, Asian, n (%)<br>Ethnicity, mixed, n (%)<br>Ethnicity, other, (%)	24 (92%)<br>1 (4%)<br>1(4%)<br>0	32 (89%)<br>1 (3%)<br>2 (5%)<br>1 (3%)	41 (93%)<br>1 (2%)<br>2 (5%)<br>0	14 (93%)<br>1 (7%)<br>0<br>0	0.97[a]	23 (100%)
Age at study, years, mean (SD)	13.6 (2.4)	12.8 (2.7)	14.0 (2.6)	14.0 (2.7)	0.20[b]	12.7 (3.1)
Age at diagnosis, years, mean (SD)	6.1 (3.9)	4.2 (2.6)	5.4 (3.5)	6.2 (3.4)	0.07[c]	
Time since diagnosis, years, mean (SD)	7.4 (3.7)	8.7 (3.1)	8.6 (3.5)	8.0 (4.1)	0.50[b]	
Time since last treatment, years, mean (SD)	5.9 (3.6)	6.3 (2.9)	7.0 (3.5)	7.1 (4.1)	0.53[b]	
BMI, kg/m^2, mean (95% CI)	20.3 (18.8, 21.9)	19.5 (18.2, 20.8)	20.5 (19.3, 21.6)	20.5 (18.5, 22.5)	0.68[d]	19.0 (17.5,20.5)
BSA, m^2, mean (95% CI)	1.5 (1.4, 1.6)	1.4 (1.3, 1.5)	1.5 (1.5, 1.6)	1.5 (1.4, 1.6)	0.34[d]	1.4 (1.3, 1.5)
Systolic BP, mmHg, mean (95% CI)	108 (104,112)	106 (102,109)	108 (105, 111)	107 (102, 112)	0.84[d]	112 (108, 116)
Diastolic BP, mmHg, mean (95% CI)	65 (62,68)	64 (61,67)	67 (65, 70)	66 (62,71)	0.56[d]	69 (65,73)
NT-proBNP, ng/L, mean (SD)	54 (30)	52 (24)	71 (36)	97 (55)	0.004[c]	
Hb, g/dL, mean (SD)	13.3 (1.2)	13.5 (0.9)	13.6 (1.0)	13.4 (0.9)	0.8[b]	
**Anthracyclines**, n	0	36 (100%)	44 (100%)	15 (100%)		
Anthracycline dose, mg/m^2, mean (SD)	0	79 (6)	152 (38)	329 (63)		
**Vinca alkaloids**, n (%)	24 (92%)	35 (97%)	41 (93%)	13 (87%)		
Vincristine, n	21	35	41	13		

Vincristine dose, mg/m^2, mean (SD)	32 (22)	30 (5)	26 (12)	21 (10)		
Vinblastine, n	3	0	0	0		
Vinorelbine, n	0	0	1	0		
**Platinum derivates**, n (%)	15 (58%)	1 (3%)	6 (14%)	5 (33%)		
Cisplatin, n	8	1	6	1		
Cisplatin dose, mg/m^2, mean (SD)	247 (155)		327 (95)			
Carboplatin, n	13	1	6	4		
Carboplatin dose, mg/m^2, mean (SD)	4734 (3581)		1919 (1372)	3199 (282)		
Radiation chest, n	0	0 (0%)	3 (7%)	2 (13%)		
EF Simpson, %, mean (95% CI)	58 (57, 60)	62 (60, 63)	60 (59, 62)	57 (55, 60)	0.01[d]	63 (61, 66)
LV-GLS, %, mean (95% CI)	-20.3 (-21.1, -19.4)	-20.3 (-20.9, -19.6)	-19.7 (-20.4, -19.1)	-18.0 (-19.0, -17.0)	0.002[d]	-21.3 (-22.2, -20.3)
LV-4C-LS, %, mean (95% CI)	-19.7 (-20.5, -18.9)	-19.9 (-20.6,-19.2)	-19.2 (-19.9, -18.6)	-18.2 (-19.2, -17.2)	0.04[d]	-21.2 (-22.1, -20.2)
Z-score LV-LS, mean (95% CI)	-0.1 (-0.6, 0.4)	0.1 (-0.4, 0.5)	-0.4 (-0.8, -0.02)	-1.1 (-1.8, -0.4)	0.03[d]	0.9 (0.2, 1.5)
RV- LS, %, mean (95% CI)	-23.6 (-24.7, -22.4)	-23.5 (-24.4, -22.6)	-22.9 (-23.8, -22.0)	-21.9 (-23.4, -20.4)	0.27[d]	-23.3 (-24.6, -22.0)
PSI-global LV, %, mean (95% CI)	2.1 (1.4, 2.8)	2.0 (1.5, 2.5)	2.2 (1.7, 2.7)	2.6 (1.8, 3.5)	0.68[d]	1.8 (1.0, 2.5)
PSI-global RV, %, mean (95% CI)	1.5 (-0.2, 3.2)	1.6 (0.5, 2.8)	2.1 (1.0, 3.2)	2.4 (0.5, 4.3)	0.84[d]	2.3 (0.7, 3.8)
Peak VO_2, ml/kg/min mean (95% CI)	38.0 (34.5, 41.4)	44.7 (41.7, 47.8)	44.7 (41.9, 47.5)	39.6 (35.0, 44.2)	<0.01[d]	48.6 (44.5, 52.6)

a= Chi-square test or Fisher`s exact test

b= classic ANOVA, post hoc Tukey

c= Welch´s ANOVA, post hoc Games-Howell

d= ANCOVA (with Sidak correction) adjusted for age, sex, BSA, echocardiographic transducer difference and treadmill location as appropriate.

CCS: childhood cancer survivor, SD: standard deviation, CI: confidence interval, BMI: body mass index, BSA: body surface area, BP: blood pressure, NT-proBNP: N-terminal pro-Brain Natriuretic peptide, Hb: hemoglobin, CI: confidence interval, EF: ejection fraction, LV-GLS: left ventricular global longitudinal strain, LV-4C-LS: left ventricular 4-chamber longitudinal strain, PSI: post systolic index, RV-LS: right ventricular longitudinal strain, VO_2: oxygen consumption, ANOVA: analysis of variance, ANCOVA: analysis of covariance
